# Population mental health improves with increasing access to treatment: evidence from a dynamic modelling analysis

**DOI:** 10.1186/s12888-022-04352-w

**Published:** 2022-11-09

**Authors:** Adam Skinner, Jo-An Occhipinti, Yun Ju Christine Song, Ian B. Hickie

**Affiliations:** 1 Brain and Mind Centre, Faculty of Medicine and Health, University of Sydney, Camperdown, Australia; 2 Computer Simulation and Advanced Research Technologies (CSART), Sydney, Australia

**Keywords:** Australia, Bayesian analysis, Mental health services, Psychological distress, System dynamics

## Abstract

**Background:**

Multiple studies indicate that the prevalence of mental disorders in high-income countries has remained stable or increased despite substantial increases in the provision of care, leading some authors to question the effectiveness of increasing access to current treatments as a means of improving population mental health.

**Methods:**

We developed a system dynamics model of mental disorder incidence and treatment-dependent recovery to assess two potential explanations for the apparent failure of increasing treatment provision to reduce mental disorder prevalence: 1) an increase in the individual-level risk of disorder onset; and 2) declining effectiveness of care resulting from insufficient services capacity growth. Bayesian Markov Chain Monte Carlo (MCMC) methods were used to fit the model to data on the prevalence of high to very high psychological distress in Australia for the period 2008–2019.

**Results:**

Estimates of yearly rates of increase in the per capita incidence of high to very high psychological distress and the proportion of patients recovering when treated indicate that the individual-level risk of developing high to very high levels of distress increased between 2008 and 2019 (posterior probability > 0.999) but provide no evidence for declining treatment effectiveness. Simulation analyses suggest that the prevalence of high to very high psychological distress would have decreased from 14.4% in 2008 to 13.6% in 2019 if per capita incidence had not increased over this period (prevalence difference 0.0079, 95% credible interval 0.0015–0.0176).

**Conclusions:**

Our analyses indicate that a modest but significant effect of increasing access to mental health care in Australia between 2008 and 2019 was obscured by a concurrent increase in the incidence of high to very high psychological distress.

**Supplementary Information:**

The online version contains supplementary material available at 10.1186/s12888-022-04352-w.

## Background

According to Global Burden of Disease study estimates for 2019, mental disorders are among the leading causes of disability globally, accounting for 14.6% of years lived with disability (ranked 2nd, after musculoskeletal disorders) and 4.9% of disability-adjusted life years [1]. At the same time, data from the World Health Organization’s World Mental Health surveys suggest that only 11.0%–60.9% of people with severe mental disorders (mean 38.9% across 17 countries) will have received any treatment in the past year [2]. Addressing this substantial ‘treatment gap’ by increasing the availability and accessibility of specialised mental health services, improving mental health training for general practitioners and allied health professionals, increasing the availability of psychotropic medications, and promoting access to care through public education programs is recognised as critical for reducing the burden of mental disorders, particularly in low- and middle-income countries, where treatment coverage is generally considerably lower than in high-income countries [3–7]. Nevertheless, multiple studies indicate that the prevalence of mental disorders in several high-income countries has remained stable or increased despite substantial increases in the provision of mental health care over the past *c*. 30 years [8–12], leading some authors to question the effectiveness of increasing access to current treatments as a means of improving population mental health [9, 10, 13].

Potential explanations for the apparent failure of increased mental health care provision to reduce the prevalence of mental disorders include increasing disorder incidence (defined here as new diagnoses plus relapse and recurrence) resulting from changing exposure to social and economic risk-modifying factors, and a decrease in the effectiveness of treatment as more people have accessed services. Where the number of people seeking help for mental disorders is increasing more rapidly than the capacity of services to provide treatment, the accessibility of services would be expected to decline (due to increasing waiting times, out-of-pocket costs, etc.), resulting in increased treatment dropout and a corresponding decline in the proportion of patients receiving minimally adequate care [2, 14]. An increase in the number of people engaging with services may result from increasing mental disorder incidence, as well as greater awareness of mental illness and available treatment options, so that the above explanations (increasing incidence and declining treatment effectiveness) are not mutually exclusive. This paper presents an analysis of the contributions of increasing disorder incidence and decreasing effectiveness of care to trends in the prevalence of high to very high psychological distress in Australia. Using a simple system dynamics model of psychological distress incidence and treatment-dependent recovery, we show that an increase in the prevalence high to very high psychological distress between 2008 and 2019 can be attributed to an increase in the individual-level risk of developing higher levels of psychological distress, and that in the absence of increasing risk, prevalence would have declined as access to treatment increased over this period.

## Methods

### Model structure and assumptions

**Fig. 1:**
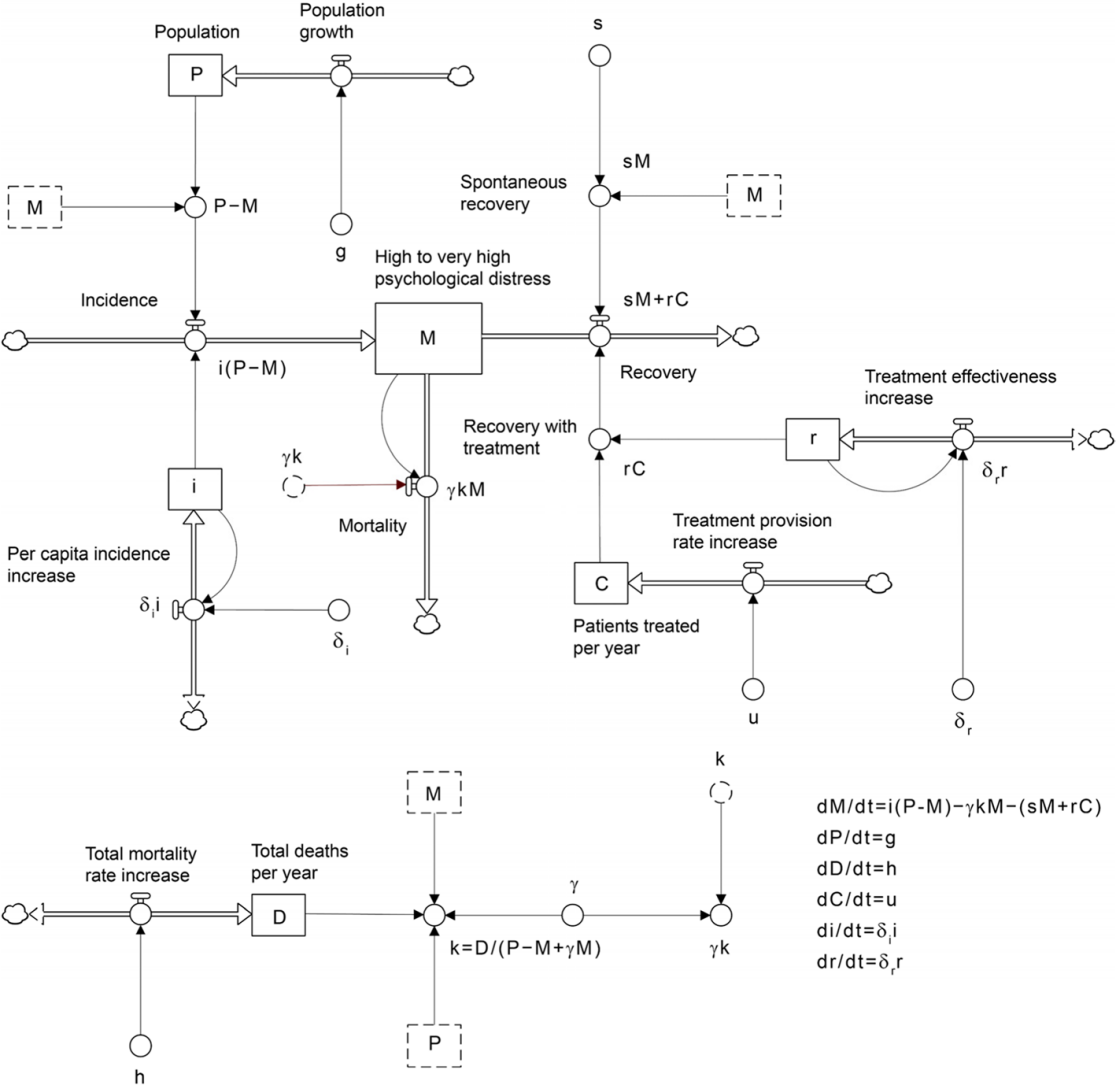
System dynamics model of psychological distress incidence and treatment-dependent recovery used for the analyses. Notation is defined in the Methods section and Table 2. Stocks (or state variables) are shown as boxes, flows as pipes with taps, causal connections (or mathematical dependencies) as arrows, and sources and sinks as clouds [15]. Symbols with dashed outlines are copies (or ‘ghosts’) of the corresponding symbols with solid outlines

Figure 1 presents the system dynamics model used for the analyses. The core of the model consists of a single stock (or state variable), labelled *M*, corresponding to the total number of people in the population currently experiencing high to very high psychological distress (defined as a K10 score of 22 or more). People with low to moderate psychological distress (K10 scores 10–21) flow into this stock at a rate (per year) equal to *i*(*P* − *M*), where *i* is the per capita rate at which people develop high to very high psychological distress per year and *P* is the total population. Mortality (due to all causes) and recovery reduce the number of people currently experiencing high to very high psychological distress at rates equal to *γkM* and *sM* + *rC*, respectively, where *k* is the per capita mortality rate per year for people with low to moderate psychological distress, *γ* is the mortality hazard ratio for people experiencing high to very high psychological distress, *s* is the per capita natural recovery rate per year, *r* is treatment effectiveness (i.e., the proportion of patients receiving treatment who recover), and *C* is the number of patients treated per year. Both the per capita incidence of high to very high psychological distress (*i*) and the proportion of patients recovering when treated (*r*) are assumed to increase (or decrease) at constant fractional rates, denoted by *δ*_*i*_ and *δ*_*r*_, respectively, that were estimated via Markov chain Mote Carlo (MCMC) simulation, as described below (see next section). Note that the incidence of high to very high psychological distress is increasing over time when *δ*_*i*_ is positive and decreasing over time when *δ*_*i*_ is negative. Similarly, positive values for *δ*_*r*_ indicate that the proportion of patients recovering when treated is increasing over time, whereas negative values indicate decreasing treatment effectiveness.

The total population (*P*), total mortality per year (equal to *k*(*P* − *M*) + *γkM*), and number of people receiving mental health care per year (*C*) are assumed to increase at constant yearly rates (*g*, *h*, and *u*, respectively), estimated from data published by the Australian Bureau of Statistics (ABS) and the Australian Institute of Health and Welfare (AIHW) (see below and Supplementary appendix 1). For simplicity, we assume that the number of people currently experiencing high to very high psychological distress (*M*) is not directly altered by migration. Thus, people entering the population via the flow labelled 'Population growth' in Fig. 1 are assumed to have low to moderate levels of psychological distress (although they may subsequently develop high to very high psychological distress), while those experiencing high to very high psychological distress only leave the stock *M* via mortality or recovery. Note also that both the number of patients treated per year and growth in the treatment provision rate are assumed to be independent of the number of people with high to very high psychological distress. Although, in principle, the number of people receiving treatment per year may be expected to depend on the prevalence of mental disorders (and therefore *M*), mental health services in Australia are generally operating at or near maximum capacity [16], so that the treatment provision rate is determined primarily by the availability and accessibility of services, not the number of people requiring care.

### Data and model fitting

Bayesian MCMC simulation [17] was used to fit the system dynamics model in Fig. 1 to Household, Income and Labour Dynamics in Australia (HILDA) Survey estimates of numbers of Australian adults (aged 18 years and above) experiencing high to very high psychological distress, population and mortality estimates published by the ABS, and estimates of numbers of people receiving publicly funded mental health care per year derived from data published by the AIHW. Details of all data sets used for model fitting are provided in Table 1. Letting *y*_*i*_(*t*) and *m*_*i*_(*t*, *θ*) denote, respectively, the observed value of data set *i* at time *t* (e.g., the HILDA Survey estimate of the number of people with high to very high psychological distress in 2017) and the corresponding model output (the modelled number of people with high to very high psychological distress in 2017) obtained for a particular set of parameter values *θ*, the likelihood for each *y*_*i*_(*t*) was calculated as *p*(*y*_*i*_(*t*)| *θ*, *β*_*i*_) = Neg-bin(*y*_*i*_(*t*)| *β*_*i*_*m*_*i*_(*t*, *θ*), *β*_*i*_), i.e., we assumed that the observed data values, *y*_*i*_(*t*), follow a negative binomial distribution with mean *m*_*i*_(*t*, *θ*) and inverse scale parameter *β*_*i*_. The parameter vector *θ* contains all of the dynamic model parameters, including the fractional rates of increase in the incidence of high to very high psychological distress and the proportion of patients recovering when treated (*δ*_*i*_ and *δ*_*r*_, respectively). Deviations of the observed data from their expected values were assumed to be independent, so the total likelihood (i.e., for all data sets and time points) is the product of the likelihoods for each *y*_*i*_(*t*).

**Table 1:** Data sets used to fit the dynamic model in Fig. 1. Note that analyses using psychological distress data from the Australian Bureau of Statistics’ National Health Survey [18] yield results qualitatively similar to those presented here for the HILDA Survey data (see Supplementary appendix 4)

Model output	Data source	Data points (*n*)	Notes
Psychological distress	Household, Income and Labour Dynamics in Australia (HILDA) Survey [19]	6 (2009, 2011, 2013, 2015, 2017, 2019)	Data on the prevalence of high to very high psychological distress (K10 scores of 22 or more) among Australian adults (aged 18 years and above) were derived from the HILDA Survey [19]. Numbers of people experiencing high to very high psychological distress, obtained by multiplying the HILDA Survey prevalence estimates by Australian Bureau of Statistics population estimates (i.e., for the same year), were used for model fitting.
Population	National demographic statistics [20]	12 (one per year for 2008–2019)	Population estimates (18 years and above, 30 June in each year) were taken directly from national demographic statistics published by the Australian Bureau of Statistics [20].
Total mortality	National mortality statistics [21]	12 (one per year for 2008–2019)	Total numbers of deaths per year (18 years and above, all causes) were taken directly from national mortality statistics published by the Australian Bureau of Statistics [21].
Patients receiving mental health care per year	*Mental Health Services in Australia* (online report) [22]	12 (one per year for 2008–2019)	National data on mental health services provision were derived from the Australian Institute of Health and Welfare’s online report *Mental Health Services in Australia* [22]. Numbers of adults (18 years and above) with high to very high psychological distress receiving Medicare-subsidised mental health services per year were obtained by multiplying total numbers of patients receiving care by estimates of the proportion of all patients receiving services who are aged 18 years and above (85.20% [22]) and the proportion of patients aged 18 years and above with high to very high psychological distress (34.45% [23]). Medicare-subsidised mental health services include Australian Government-funded services provided by general practitioners, psychiatrists, and psychologists and other allied health professionals.

MCMC simulation was performed using Stan ver. 2.21.5 [24]. Prior distributions specified for the dynamic model parameters in *θ* are described in Table 2. We ran four Markov chains in parallel for 4000 iterations and used the final 2000 iterations from each chain (8000 samples combined) for posterior inference (i.e., the initial half of each chain was discarded as warmup). Trace plots and marginal posterior distributions for all model parameters are presented in Supplementary appendix 2. Model fit was assessed for each of the data sets used in our analyses via posterior predictive simulation, using the *χ*^2^ discrepancy as a measure of lack of fit (see Supplementary appendix 2).

**Table 2:** Prior distributions specified for the dynamic model parameters

Parameter	Symbol	Prior distribution^a^	Notes
Initial prevalence of high to very high psychological distress	*M*_0_/*P*_0_	normal(0.1464, 0.0293) T[0, 1]	Normal prior (truncated at 0 and 1) with mean 0.146 and standard deviation equal to 20% of the mean (0.029). Data from the Household, Income and Labour Dynamics in Australia (HILDA) Survey indicate that the prevalence of high to very high psychological distress among Australian adults (aged 18 years and above) was 14.80% in 2007 and 14.48% in 2009 [19]. The specified prior distribution assumes that the prevalence of high to very high psychological distress at the start of 2008 was near to the midpoint of the 2007 and 2009 HILDA Survey estimates.
Initial population	*P*_0_	normal(16167328, 3233466) T[0,]	Normal prior (truncated at 0) with mean equal to a linear regression estimate of the Australian adult population at the start of 2008 and standard deviation equal to 20% of the mean.
Population increase per year	*g*	normal(308538, 61708)	Normal prior with mean equal to the slope of a linear regression line fitted to Australian Bureau of Statistics population data for the period 2008–2019 [20] and standard deviation equal to 20% of the mean.
Initial total mortality per year	*D*_0_	normal(136840, 27368) T[0,]	Normal prior (truncated at 0) with mean equal to a linear regression estimate of total adult mortality per year at the start of 2008 and standard deviation equal to 20% of the mean.
Total mortality increase per year	*h*	normal(2228.7, 445.7)	Normal prior with mean equal to the slope of a linear regression line fitted to Australian Bureau of Statistics mortality data for the period 2008–2019 [21] and standard deviation equal to 20% of the mean.
Mortality hazard ratio	*γ*	lognormal(−0.7733, 0.4701)	Lognormal prior applied to *γ* − 1 with mode 0.37 (*γ* = 1.37) and 95th percentile equal to 1 (i.e., the probability that *γ* exceeds 2 is 0.05). Russ et al. [25] reported mortality hazard ratios of 1.37 (95% confidence interval 1.23–1.51) and 1.67 (1.41–2.00) for people with GHQ-12 psychological distress scores of 4–6 and 7–12, respectively. GHQ-12 scores for participants in the 1997 Australian National Survey of Mental Health and Wellbeing with K10 scores of 25 or more were typically above 4 (mean *c*. 4.81 [26]), so we assume that most people with high to very high psychological distress (according to the K10 scale) would have GHQ-12 scores in the range 4–12. As the lognormal distribution has support on the positive real numbers only, the specified prior assumes that *γ* is greater than 1 (i.e., the per capita mortality rate for people experiencing high to very high psychological distress is assumed to be higher than that for people with lower levels of psychological distress).
Initial per capita incidence of high to very high psychological distress	*i*_0_	normal(0, 0.5) T[0,]	Non-informative normal prior (truncated at 0) with mean 0 and standard deviation 0.5.
Fractional increase in per capita incidence per year	*δ*_*i*_	normal(0, 0.5)	Non-informative normal prior distribution with mean 0 and standard deviation 0.5.
Natural (or spontaneous) recovery rate	*s*	lognormal(−1.1315, 0.2024)	Lognormal prior with mode 0.31 and 95th percentile equal to 0.45 (i.e., the prior probability that *s* exceeds 0.45 is 0.05). The specified distribution assumes that the proportion of people with high to very high psychological distress recovering spontaneously per year (i.e., without receiving treatment) is similar to that for primary care patients with undiagnosed (and therefore untreated) major depressive disorders, estimated to be 31.0% [27].
Initial services provision rate (i.e., patients treated per year)	*C*_0_	normal(305315, 61063) T[0,]	Normal prior (truncated at 0) with mean equal to a linear regression estimate of the number of people receiving treatment per year at the start of 2008 and standard deviation equal to 20% of the mean.
Services provision rate increase per year	*u*	normal(43696, 8739)	Normal prior with mean equal to the slope of a linear regression line fitted to services provision rate estimates for the period 2008–2019 (see Table 1) and standard deviation equal to 20% of the mean.
Initial proportion of patients recovering when treated (i.e., treatment effectiveness)	*r*_0_	normal(0.1845, 0.0369) T[0, 1]	Normal prior (truncated at 0 and 1) with mean 0.18 and standard deviation equal to 20% of the mean. The specified distribution assumes that the initial proportion of patients with high to very high psychological distress recovering when treated is close to the sustained response rate for patients with major depressive disorders receiving combined psychotherapy and pharmacotherapy (estimated to be 45% [28]) multiplied by the estimated proportion of patients accessing mental health services who receive minimally adequate treatment (41% [29]).
Fractional increase in treatment effectiveness per year	*δ*_*r*_	normal(0, 0.0125)	Normal prior with mean 0 and standard deviation 0.0125. Australian Institute of Health and Welfare data on the provision of Medicare-subsidised mental health services indicate that the mean number of services provided per patient declined from 5.14 in 2008 to 4.54 in 2019 [22]. Although this decline does not necessarily imply decreasing quality of care (e.g., it may simply reflect declining disorder severity among patients presenting to services), the fractional rate of decline in the mean number of services per patient, equal to 1.14% per year, indicates, very roughly, the maximum plausible rate of decay in treatment effectiveness. No assumption is made about the direction of change (if any) in the proportion of patients recovering with treatment (the specified prior is symmetrical about 0); however, we assume that the absolute value of *δ*_*r*_ is no more than *c*. 3.75% per year, or roughly 3.3 times the fractional rate of decline in the mean number of services per patient over the study period.

^a^ Prior distributions are given using Stan notation [24]

## Results

**Fig. 2:**
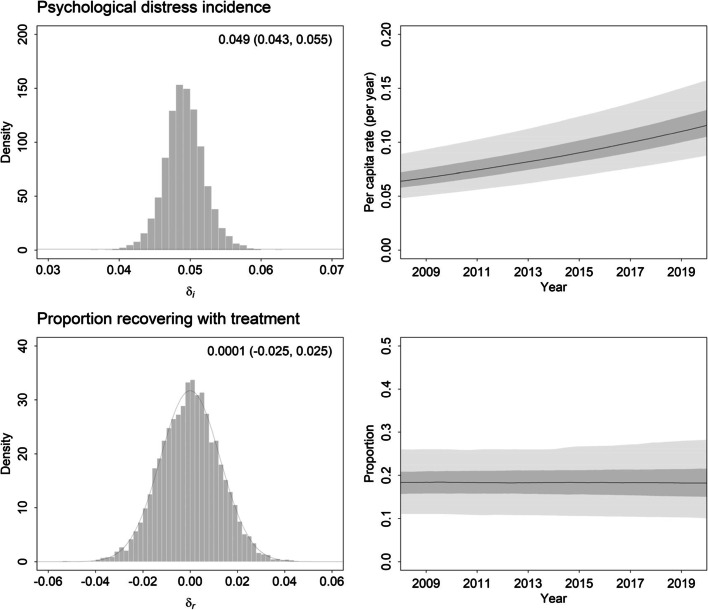
Left panels. Marginal posterior distributions estimated for the fractional rate of increase in the per capita incidence of high to very high psychological distress (*δ*_*i*_) and the fractional rate of increase in treatment effectiveness (*δ*_*r*_). Median estimates and 95% credible intervals are shown in the top right corner of each panel. Prior distributions are plotted as smooth curves. The close similarity of the posterior and prior distributions for *δ*_*r*_ indicates that the available data provide no evidence for declining (or increasing) treatment effectiveness (the prior is symmetrical about 0). Right panels. Modelled trajectories for the per capita incidence of high to very high psychological distress (*i*) and the proportion of patients recovering with treatment (*r*) over the period 2008 to 2020. Pointwise 50 and 95% credible intervals (calculated from the output of 10^3^ simulations, each using a randomly selected parameter vector *θ* sampled in the Markov chain Monte Carlo analysis) are indicated with dark grey shading and light grey shading, respectively

**Fig. 3:**
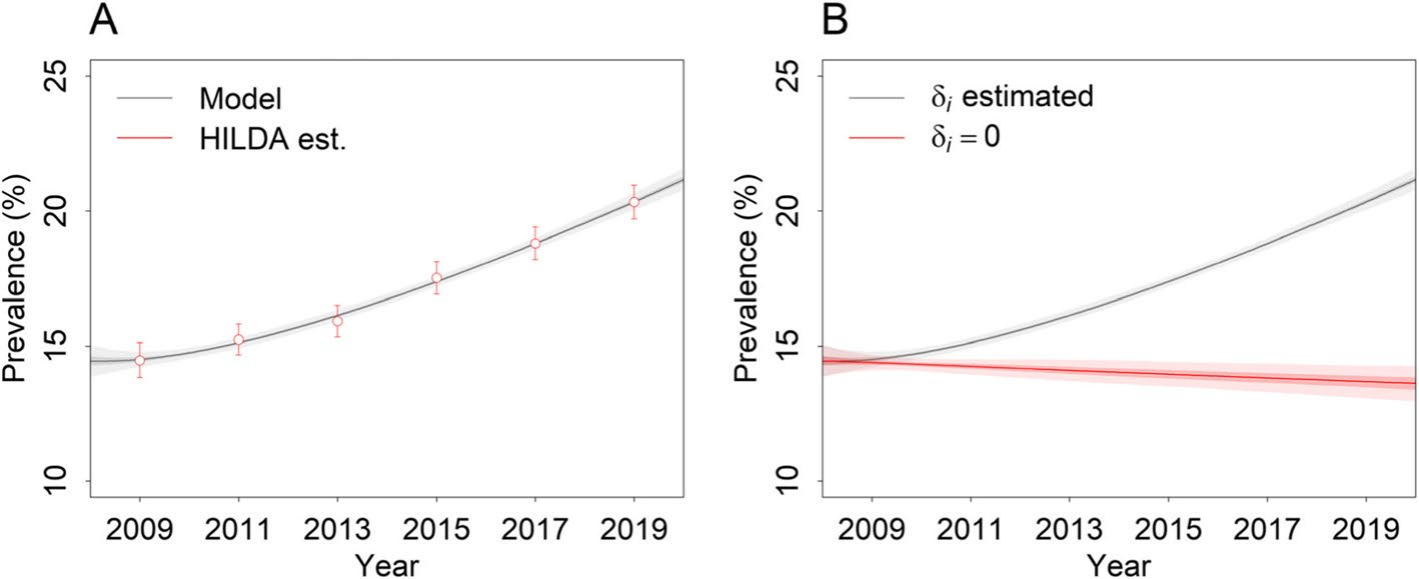
**A** Estimates of the prevalence of high to very high psychological distress among Australian adults (18 years and above) over the period 2008 to 2020 derived from the Household, Income and Labour Dynamics in Australia (HILDA) Survey (red open circles with 95% confidence intervals; see ref. [19]) and the system dynamics model (dark grey line, obtained assuming median parameter estimates). Pointwise 50 and 95% credible intervals (calculated from the output of 10^3^ simulations, each using a randomly selected parameter vector *θ* sampled in the Markov chain Monte Carlo analysis) are indicated with dark grey shading and light grey shading, respectively. **B** Prevalence of high to very high psychological distress simulated under a counterfactual scenario in which per capita incidence remains constant over time (*δ*_*i*_ is set to 0; red line). The model-based estimates from panel **A** (where *δ*_*i*_ is estimated from the HILDA Survey data) are also plotted for comparison (dark grey line). Pointwise 50 and 95% credible intervals are indicated with dark shading and light shading, respectively

Marginal posterior distributions inferred for the fractional rate of increase in the per capita incidence of high to very high psychological distress and the fractional rate of increase in treatment effectiveness (*δ*_*i*_ and *δ*_*r*_, respectively) are presented in Fig. 2. The posterior probability that the yearly increase in the per capita incidence of high to very high psychological distress exceeds 0 is very close to 1 (more than 99.98% of the marginal posterior distribution lies above 0), indicating that the individual-level risk of developing high to very high psychological distress increased over the period from 2008 to 2019. In contrast, there is no evidence for a decline in treatment effectiveness between 2008 and 2019; the posterior probability that the yearly increase in the proportion of patients recovering with treatment is negative is only 0.497 (i.e., it is nearly equally probable that treatment effectiveness increased over the study period). Panels A and B of Fig. 3 show, respectively, the fit of the system dynamics model to the psychological distress data and the prevalence of high to very high psychological distress under a counterfactual scenario in which per capita incidence remains constant over time (*δ*_*i*_ is set to 0). The results in panel B indicate that the prevalence of high to very high psychological distress would have decreased by 0.79 percentage points (95% credible interval [CrI] 0.15–1.76) between 2008 and 2019 if the individual-level risk of developing high to very high psychological distress had not increased over this period. Multiplying this prevalence decrease (0.0079) by the adult population in 2019, we obtain an estimate of 154,802 (95% CrI 29,984–345,276) fewer people with high to very high psychological distress, corresponding to 5.47% (95% CrI 1.06%–12.2%) of the number of people who would have been experiencing high to very high psychological distress if prevalence had remained constant (i.e., at the 2008 value of 14.4%).

**Fig. 4:**
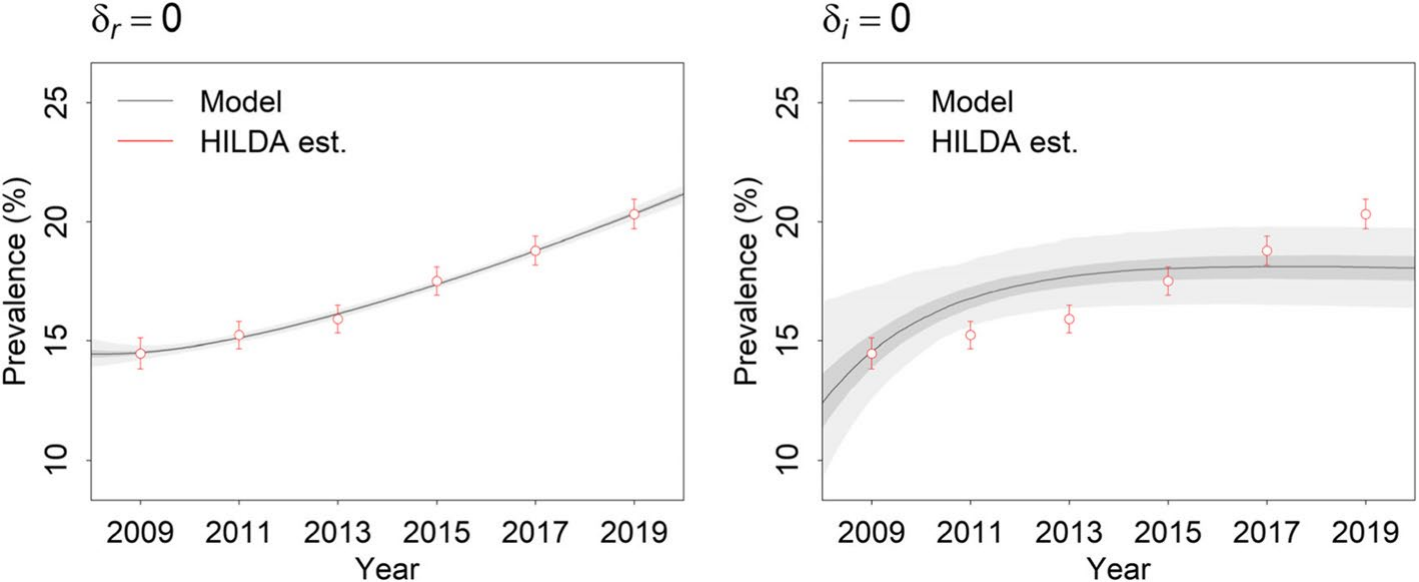
Estimates of the prevalence of high to very high psychological distress among Australian adults (18 years and above) derived from the Household, Income and Labour Dynamics in Australia (HILDA) Survey (red open circles with 95% confidence intervals) and from constrained models in which the fractional rates of increase in the per capita incidence of high to very high psychological distress and treatment effectiveness (*δ*_*i*_ and *δ*_*r*_, respectively) are assumed to equal 0 (dark grey lines). Pointwise 50 and 95% credible intervals are indicated with dark grey shading and light grey shading, respectively

Figure 4 presents a comparison of models in which the fractional rates of increase in the per capita incidence of high to very high psychological distress and the proportion of patients recovering when treated are assumed to equal 0 (note that in this case we have fitted the constrained models to the data, unlike in the counterfactual simulation presented in panel B of Fig. 3, where values for all parameters except *δ*_*i*_ are derived from the unconstrained model analysis). Allowing per capita incidence to increase over time (while holding treatment effectiveness constant) yields a markedly better fit to the psychological distress data than allowing treatment effectiveness to decline (where per capita incidence is held constant), consistent with the results for the unconstrained model presented in Fig. 2.

## Discussion

The modelling results presented above indicate that an observed increase in the prevalence of high to very high psychological distress in Australia between 2008 and 2019 (see Fig. 3, panel A) can be explained by an increase in the per capita rate at which people with low to moderate psychological distress develop more severe anxiety or depressive symptoms. Our results provide no evidence for a decline in the proportion of patients recovering when treated (an increase in treatment effectiveness is equally probable), so that the per capita treatment-dependent recovery rate may be assumed to have increased significantly with the substantial increase in access to mental health care over the study period (10.6% of the population accessed services in 2019, compared to 4.79% in 2008; see Supplementary appendix 3) [22]. The simulation results presented in panel B of Fig. 3 suggest that the prevalence of high to very high psychological distress would have decreased from 14.4% in 2008 to 13.6% in 2019 if the per capita incidence of high to very high distress had been stable over this period. As the per capita spontaneous recovery rate is assumed to be constant and per capita mortality declines only slightly (see Supplementary appendix 1), this decrease in prevalence, which equates to a 5.47% reduction in the number of people with high to very high psychological distress in 2019, is due primarily to an increase in treatment-dependent recovery. Accordingly, our analyses provide no support for the proposal that limited treatment effectiveness (reflected in the low value of *r*; see Table 2 and Fig. 2) severely restricts the potential impact of increasing access to care on population mental health [9]. Rather, we conclude that the increase in treatment provision in Australia from 2008 to 2019 was simply insufficient to offset a concurrent increase in the incidence of high to very high psychological distress.

Previous studies that have examined the potential for changing exposure to risk-modifying factors to obscure (or ‘mask’) a significant effect of increasing treatment provision on population mental health have generally concluded that there is no evidence for an increase in the individual-level risk of developing mental disorders that could explain stable or increasing disorder prevalence [9, 13]. Jorm et al. [9] noted that in Australia, the impacts of recent natural disasters and the global financial crisis have been either localised or relatively small, while exposure to potentially traumatic events (including interpersonal violence, life-threatening accidents, etc.) and poor physical health has remained constant or declined over time. Nevertheless, there are a number of economic and social trends that could plausibly have contributed to an increase in the per capita incidence of high to very high psychological distress in Australia from 2008 to 2019, including increasing underemployment and insecure employment [30], increasing household debt [19], a decline in the frequency of social interaction [31], and increasing loneliness [32]. Analyses of high-quality panel data (e.g., from the HILDA Survey) [19] examining the potential impacts of these (and other) trends on the individual-level risk of developing mental disorders are needed before changing exposure to social and economic risk-modifying factors can be excluded as a cause of increasing disorder prevalence in Australia.

For the purpose of the analyses presented here, incidence is defined to include the onset of both initial episodes of high to very high psychological distress and recurrent episodes (see Background section); people leaving the stock *M* via the recovery outflow (where recovery corresponds to a decrease in K10 score to less than 22) are assumed to return via the incidence inflow if they re-develop symptoms (see Fig. 1). Consequently, incidence will depend not only on exposure to economic and social factors that modify the risk of onset of depressive and anxiety symptoms, but also on the effectiveness of mental health care provided during and after an initial episode of high to very high psychological distress. Ormel et al. [33] recently proposed that an absence of evidence for a decline in the prevalence of depressive disorders accompanying increases in treatment efficacy and availability over the past 40 years (the ‘treatment-prevalence paradox’) may be partially explained by significantly lower effectiveness of interventions for preventing relapse and recurrence in real-world clinical practice than in randomised controlled trials. Assuming these interventions typically have some real-world effect (i.e., they are not completely ineffective), however, it is unclear why a substantial increase in treatment provision should not be expected to reduce disorder prevalence at least marginally, unless prevalence is increasing due to changing exposure to risk-modifying factors or treatment effectiveness is declining (this also applies to a similar explanation focussing on acute-phase treatments; see ref. [33]). Although a decline in the effectiveness of interventions for preventing relapse and recurrence could in principle explain an increase in incidence in our model (since incidence includes the onset of recurrent episodes of psychological distress), this decline would have to occur despite no change in the effectiveness of acute-phase treatment (equal to *r* in our model; see Fig. 2).

### Limitations

There are several important limitations of our analyses that should be pointed out. First, we have only modelled the prevalence of high to very high psychological distress (consistent with the focus of previous studies) [9], yet a substantial proportion of people accessing mental health services will be experiencing less severe symptoms (in 2007–08, 65.6% of Australian adults consulting a mental health professional in the past year had K10 scores of 21 or lower) [23]. Effective treatment of mild to moderate mental disorders has the potential not only to significantly reduce the burden of mental illness directly (since most people with mental disorders will have mild to moderate symptoms), but also to reduce the prevalence of severe mental disorders by preventing disease progression [34]. The analyses presented here provide no indication of the impact of increasing provision of care on the prevalence of mild to moderate psychological distress, and do not permit an assessment of the possibility that treatment effectiveness has declined for patients with mild to moderate symptoms (due to insufficient services capacity; see Background section), resulting in an increase in the incidence of more severe psychological distress. Second, we have assumed that the sensitivity of the K10 scale remained unchanged over the study period; however, it is possible that people have become more inclined to disclose symptoms of distress over time, leading to an apparent (rather than actual) increase in the prevalence of high to very high psychological distress (see, however, ref. [35]). And third, our analyses focus exclusively on the prevalence of psychological distress in Australia, so that additional studies are needed to determine if our conclusions apply to other countries where increasing access to care appears to have had minimal impact on mental disorder prevalence.

## Conclusion

The dynamic modelling analyses presented here indicate that an increase in the prevalence of high to very high psychological distress in Australia from 2008 to 2019 is attributable to an increase in the per capita incidence of higher levels of distress rather than declining treatment effectiveness. While the causes of this increase in incidence are unclear, there are several relatively recent economic and social trends that could plausibly explain an increase in the individual-level risk of developing more severe anxiety and depressive symptoms, including increasing underemployment, declining employment security, increasing household debt, a decline in the frequency of social interaction, and increasing loneliness. Significantly, our simulation results indicate that if the per capita incidence of high to very high psychological distress had been stable over the study period, increasing treatment-dependent recovery associated with a substantial increase in access to mental health care would have produced a modest but significant decrease in the proportion of people with high to very high K10 scores. Accordingly, while substantial progress in reducing the burden of mental disorders may be assumed to depend on improving the effectiveness of mental health care and increased investment in prevention programs (addressing the ‘quality gap’ and the ‘prevention gap’) [9], the results of our analyses provide no evidence that increasing access to currently available treatments will be any less critical for improving population mental health. Addressing the substantial and persistent ‘treatment gap’ for mental disorders should remain a global public health priority.

## Supplementary Information

**Additional file 1.**

## Data Availability

The datasets used and/or analysed during the current study are available from the corresponding author on reasonable request.

## Abbreviations

ABS: Australian Bureau of Statistics
AIHW: Australian Institute of Health and Welfare
CrI: credible interval
HILDA: Household, Income and Labour Dynamics in Australia
K10: Kessler 10
MCMC: Markov chain Monte Carlo
