# Supplementary material for: Population mental health improves with increasing access to treatment: evidence from a dynamic modelling analysis
Source: BMC Psychiatry. 2022 Nov 9;22:692. doi: 10.1186/s12888-022-04352-w (PMC9644460; doi:10.1186/s12888-022-04352-w)
Supplement: Supplementary file 1 — Additional file 1. [file 12888_2022_4352_MOESM1_ESM.docx]

Supplementary information. Population mental health improves with increasing access to treatment: evidence from a dynamic modelling analysis

Adam Skinner^1,*^, Jo-An Occhipinti^1,2^, Yun Ju Christine Song^1^, Ian B. Hickie^1^

^1^ Brain and Mind Centre, Faculty of Medicine and Health, University of Sydney, Sydney, Australia; ^2^ Computer Simulation and Advanced Research Technologies (CSART), Sydney, Australia

^*^ Corresponding author. Email: adam.skinner@sydney.edu.au. Address: Level 4, Building M02C, 94 Mallet Street, Camperdown, NSW 2050, Australia. Phone: +61 450 458 201.

Supplementary appendix 1

Comparison of dynamic model outputs with empirical data used for model fitting (see Table 1 of the paper)


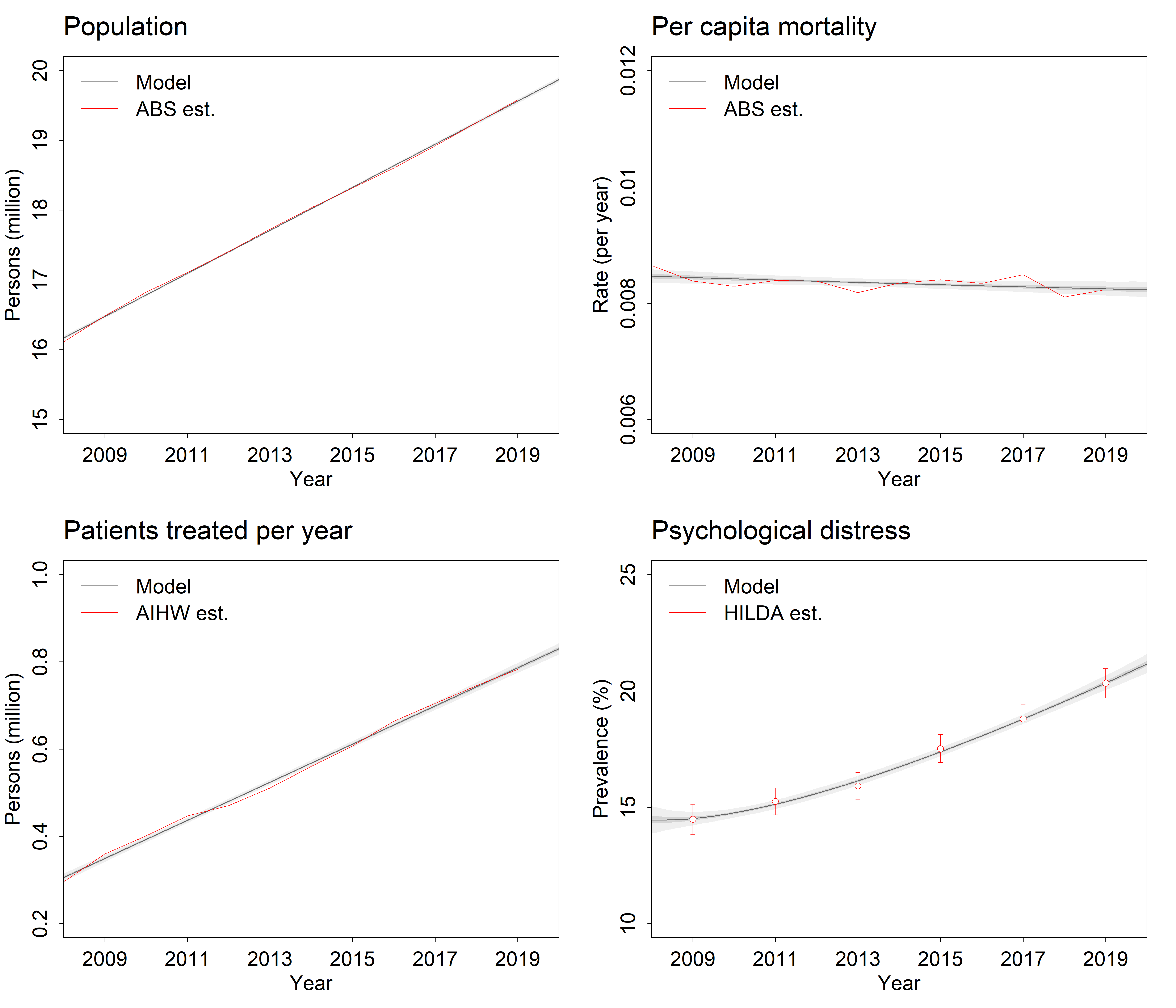


Figure S1. Comparison of dynamic model results (grey lines, obtained assuming median parameter estimates) with the empirical data used for model fitting (see Table 1 of the paper). Pointwise 50% and 95% credible intervals for the model outputs (calculated from 10^3^ simulations, each of which used a randomly selected parameter vector $\theta$ sampled in the Markov chain Monte Carlo analysis) are indicated with dark grey shading and light grey shading, respectively. Abbreviations: ABS — Australian Bureau of Statistics; AIHW — Australian Institute of Health and Welfare; HILDA — Household, Income and Labour Dynamics in Australia Survey.

Supplementary appendix 2

Markov chain Monte Carlo (MCMC) simulation methods

Markov chain Monte Carlo (MCMC) simulation was performed using Stan ver. 2.21.2 (Carpenter et al., 2017). Prior distributions for the dynamic model parameters in $\theta$ are in Table 2 of the paper. We specified non-informative exponential prior distributions with means of 10^4^ for the negative binomial distribution inverse scale parameters $\beta_{i}$ (see Methods section of the paper). Four Markov chains, each initialised with parameter values sampled from the joint prior distribution, were run in parallel for 4000 iterations; we used the final 2000 iterations from each chain (8000 samples combined) for posterior inference (i.e., the initial half of each chain was discarded as warmup; Gelman et al., 2014). Potential scale reduction factors ($\hat{R}$) calculated for all parameters were less than 1.01, indicating approximate convergence to the posterior distribution (see Gelman et al., 2014). Trace plots and marginal posterior distributions for all model parameters are shown in figure S2. Model fit was assessed for each of the data sets used in our analyses (see Table 1 of the paper) via posterior predictive simulation, using the $\chi^{2}$ discrepancy as a measure of lack of fit (see Gelman et al., 1996). Posterior predictive *p*-values range from 0.455−0.646, indicating acceptable overall fit for all data sets (see figure S3).

References

Carpenter, B., Gelman, A., Hoffman, M. D., Lee, D., Goodrich, B., Betancourt, M., Brubaker, M. A., Guo, J., Li, P., Riddell, A., 2017. Stan: a probabilistic programming language. J. Stat. Softw. 76 (1), 1‒32.

Gelman, A., Carlin, J. B., Stern, H. S., Dunson, D. B., Vehtari, A., Rubin, D. B., 2014. Bayesian data analysis. CRC Press, Boca Raton.

Gelman, A., Meng, X.-L., Stern, H., 1996. Posterior predictive assessment of model fitness via realized discrepancies. Stat. Sin. 6, 733−807.


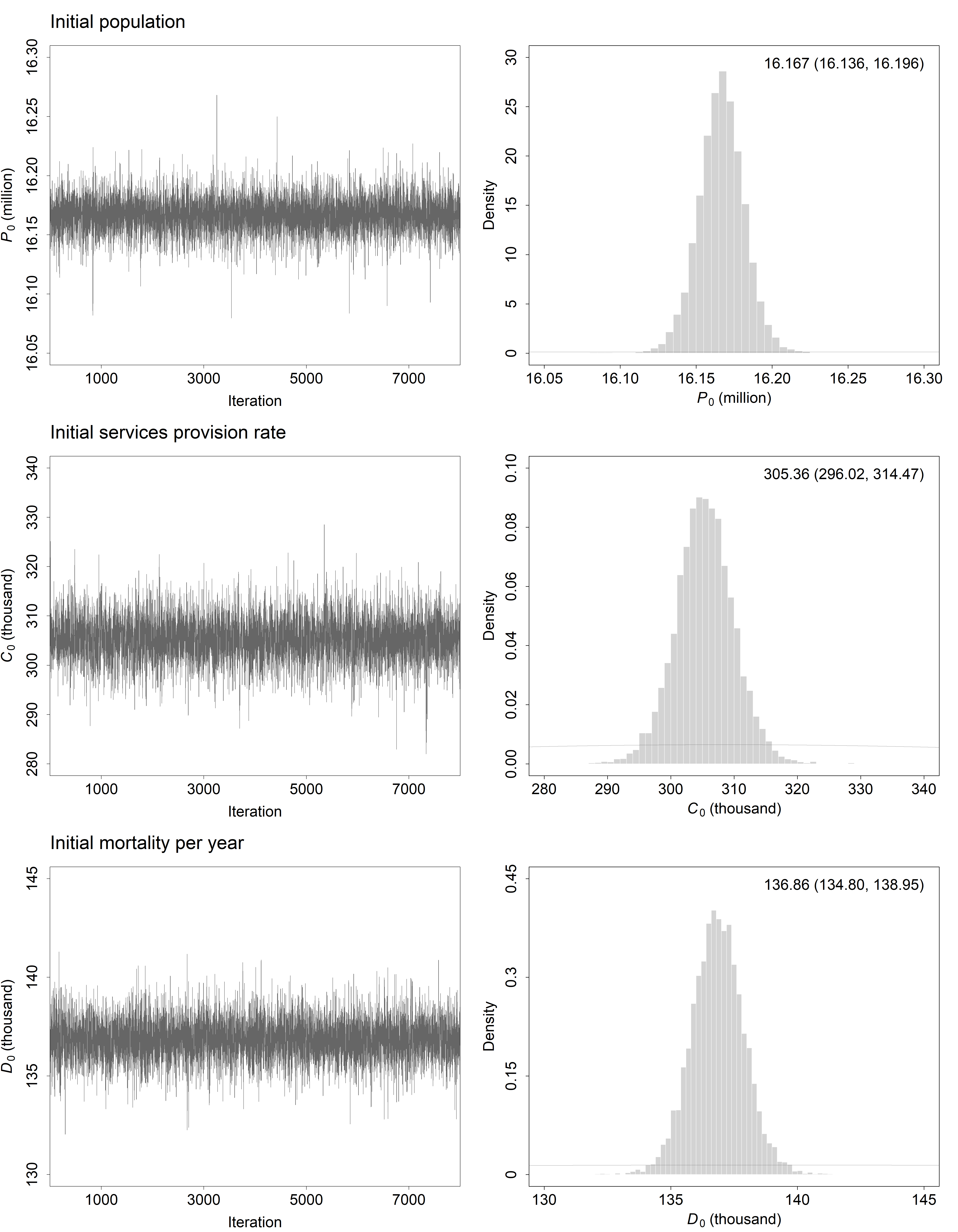


Figure S2. Markov chain Monte Carlo analysis results. Left panels. Trace plots. Post-warmup samples from the four separate Markov chains are concatenated in each plot (i.e., samples 1‒2000 are from the first chain, samples 2001‒4000 are from the second chain, etc.). Right panels. Marginal posterior distributions. Mean estimates and 95% credible intervals are shown in the top right corner of each panel. Prior distributions are plotted as smooth curves.


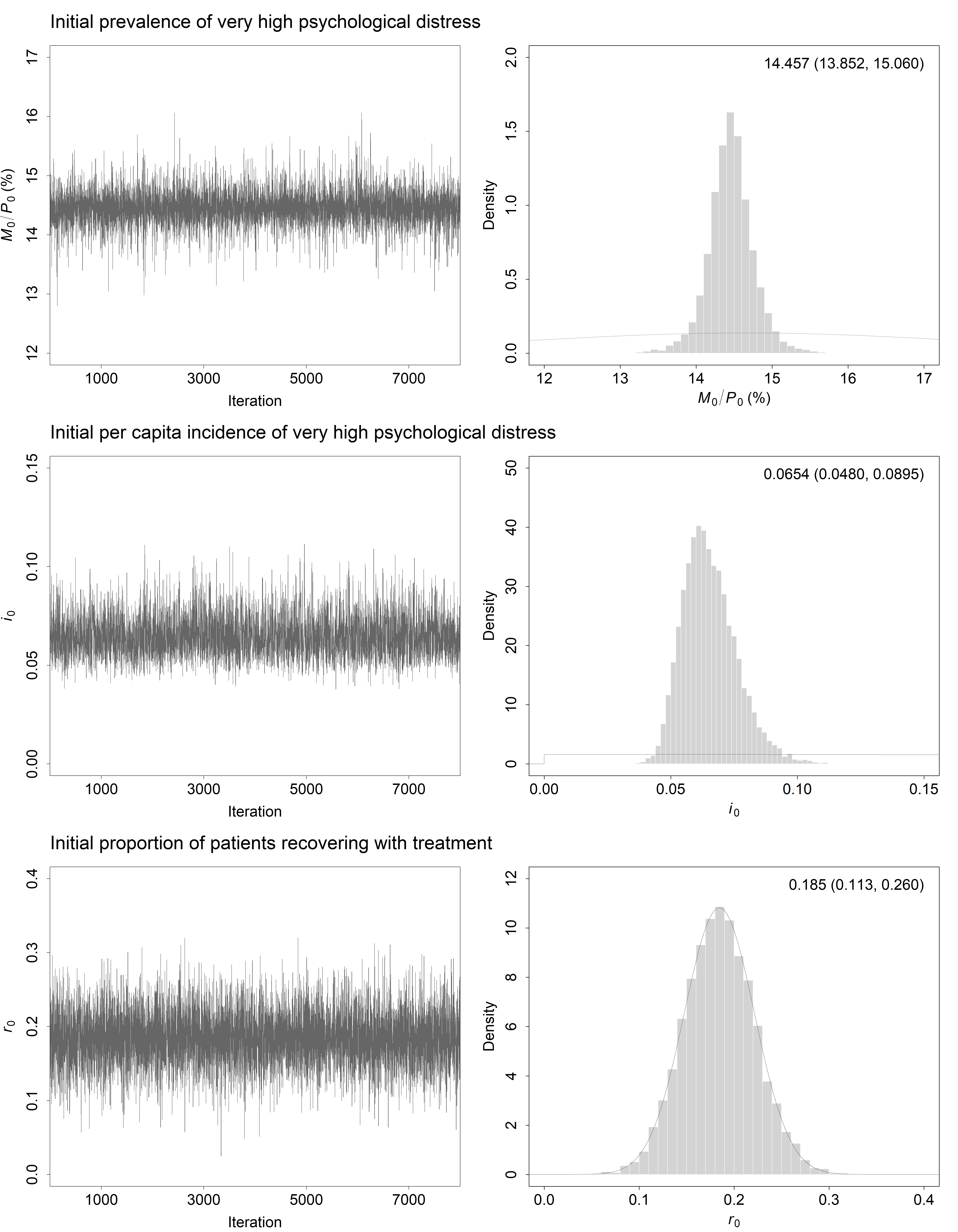


Figure S2, cont’d.


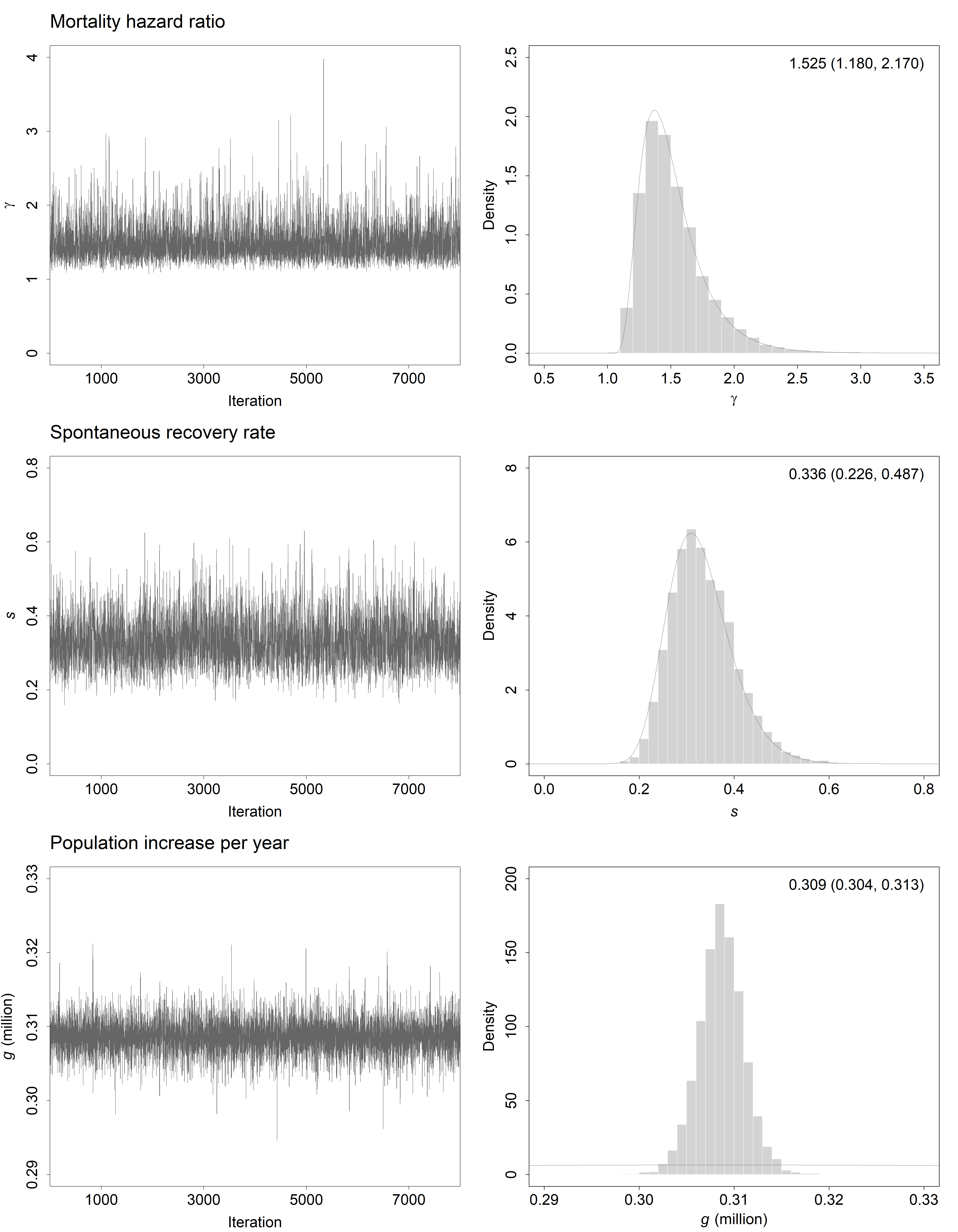


Figure S2, cont’d.


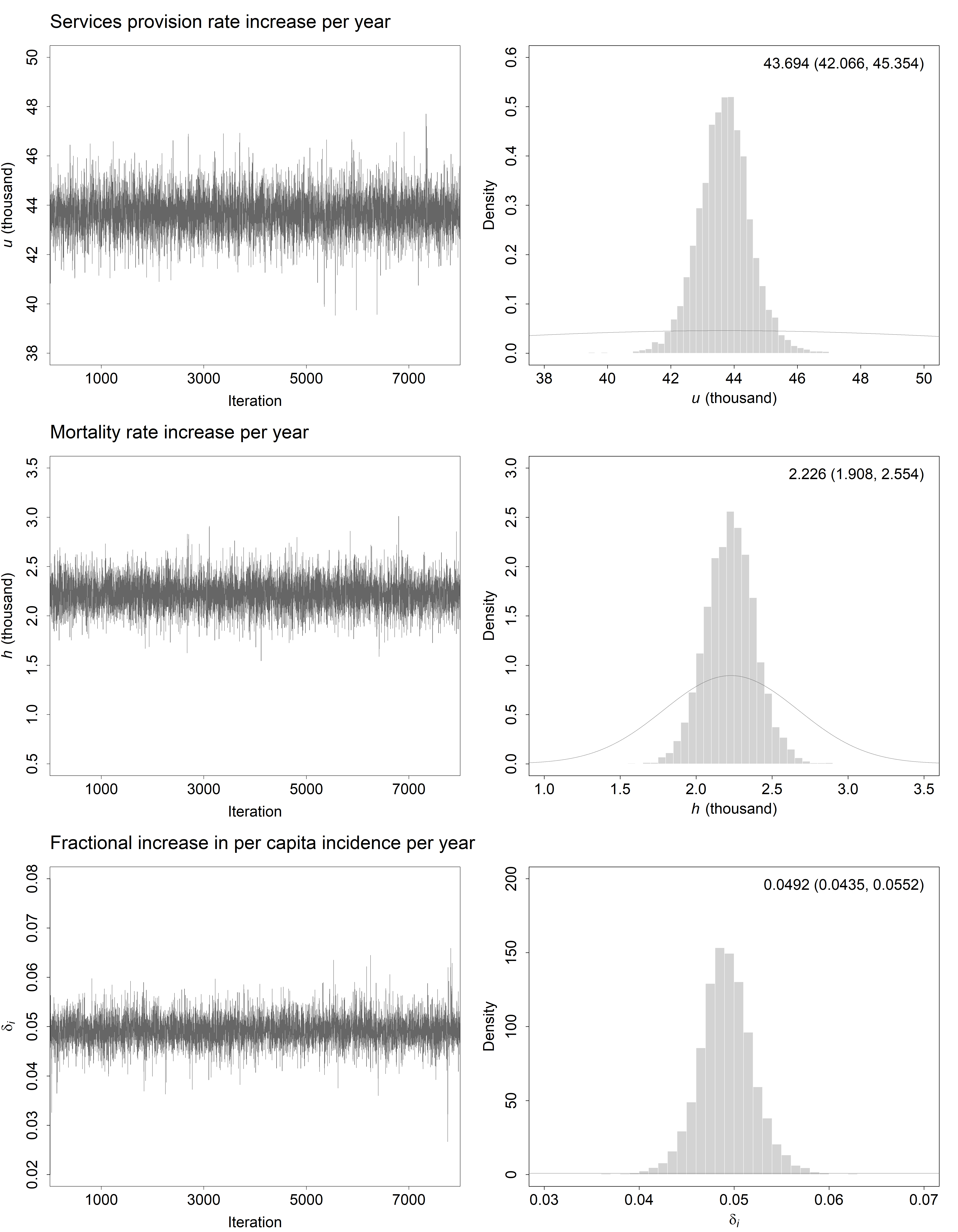


Figure S2, cont’d.


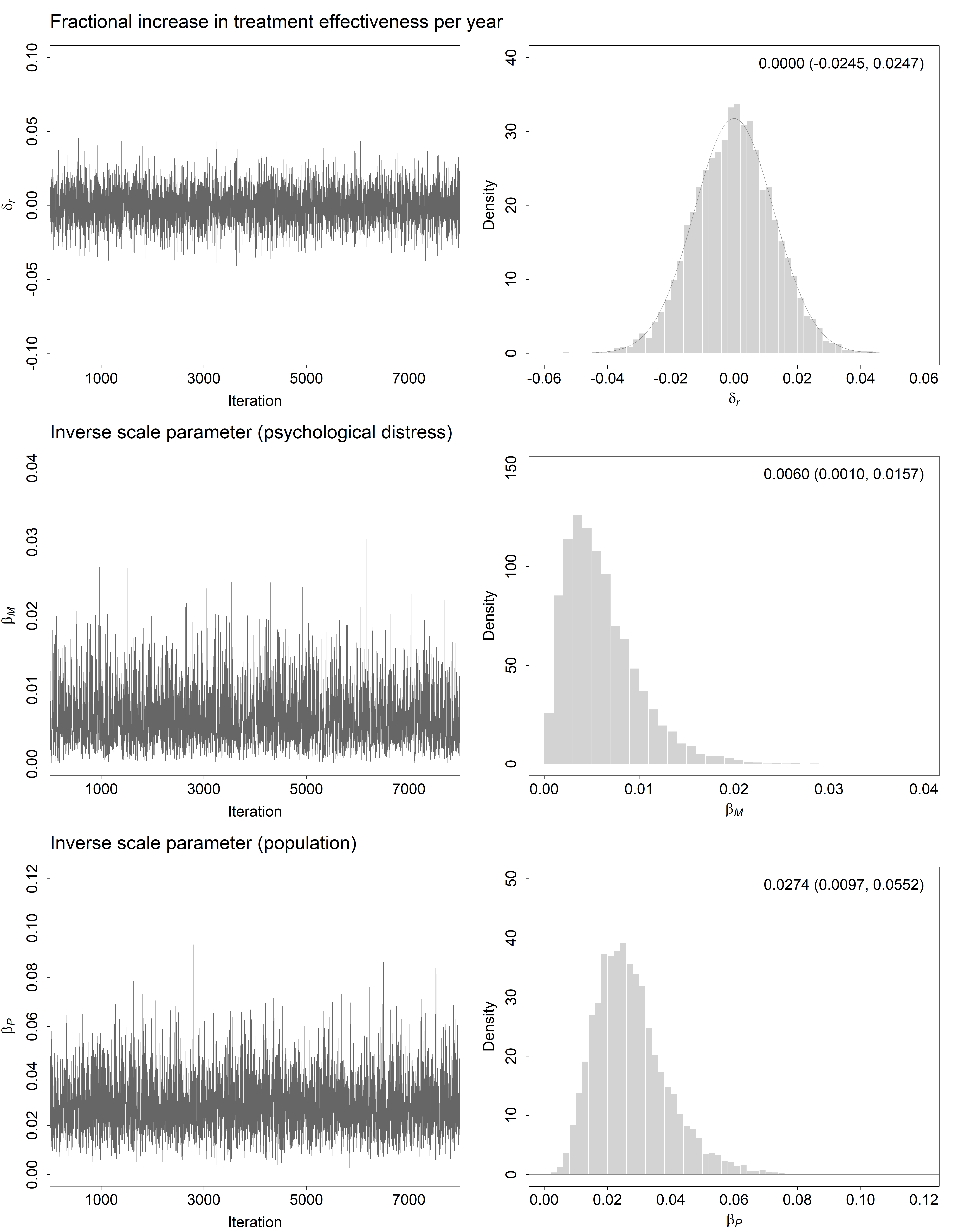


Figure S2, cont’d.


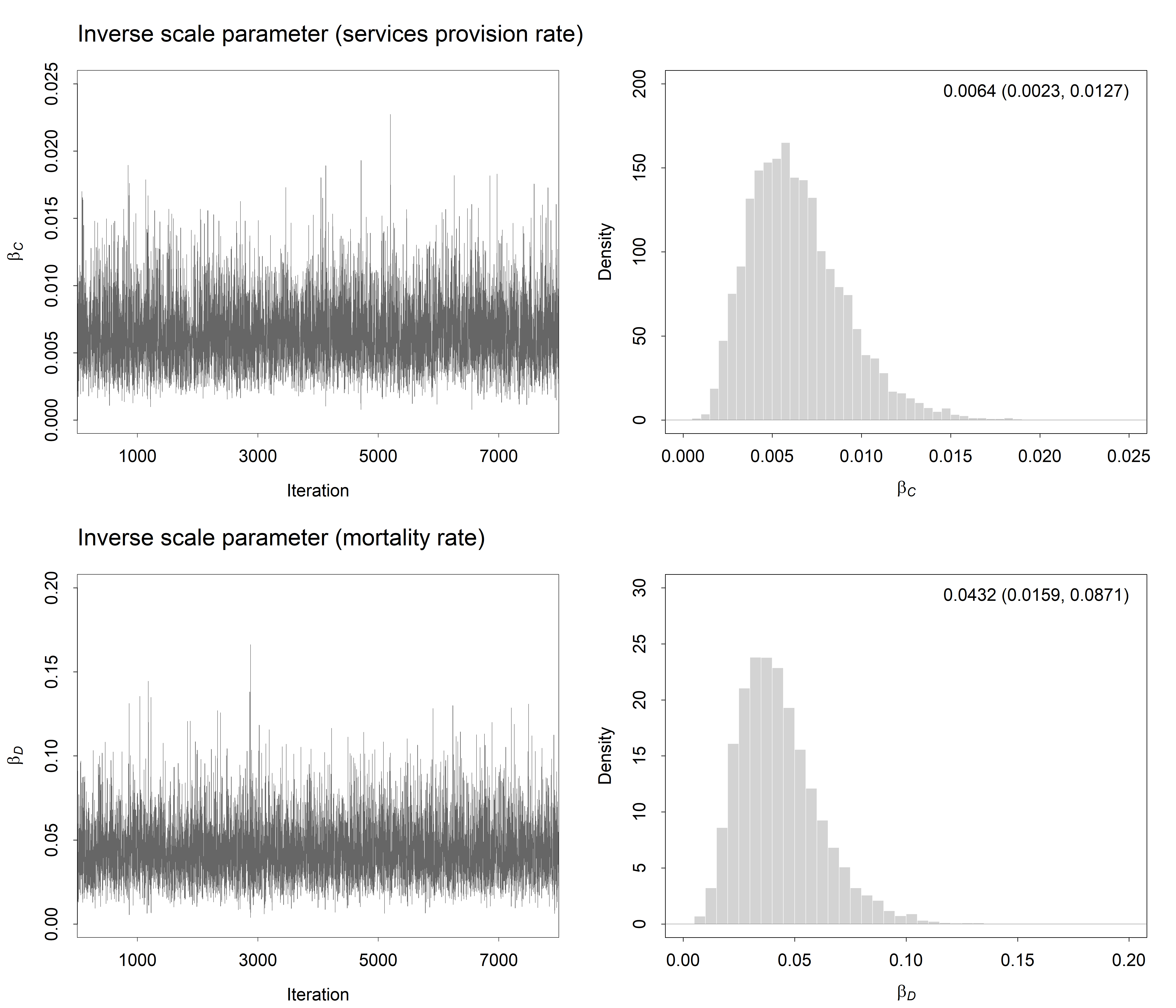


Figure S2, cont’d.


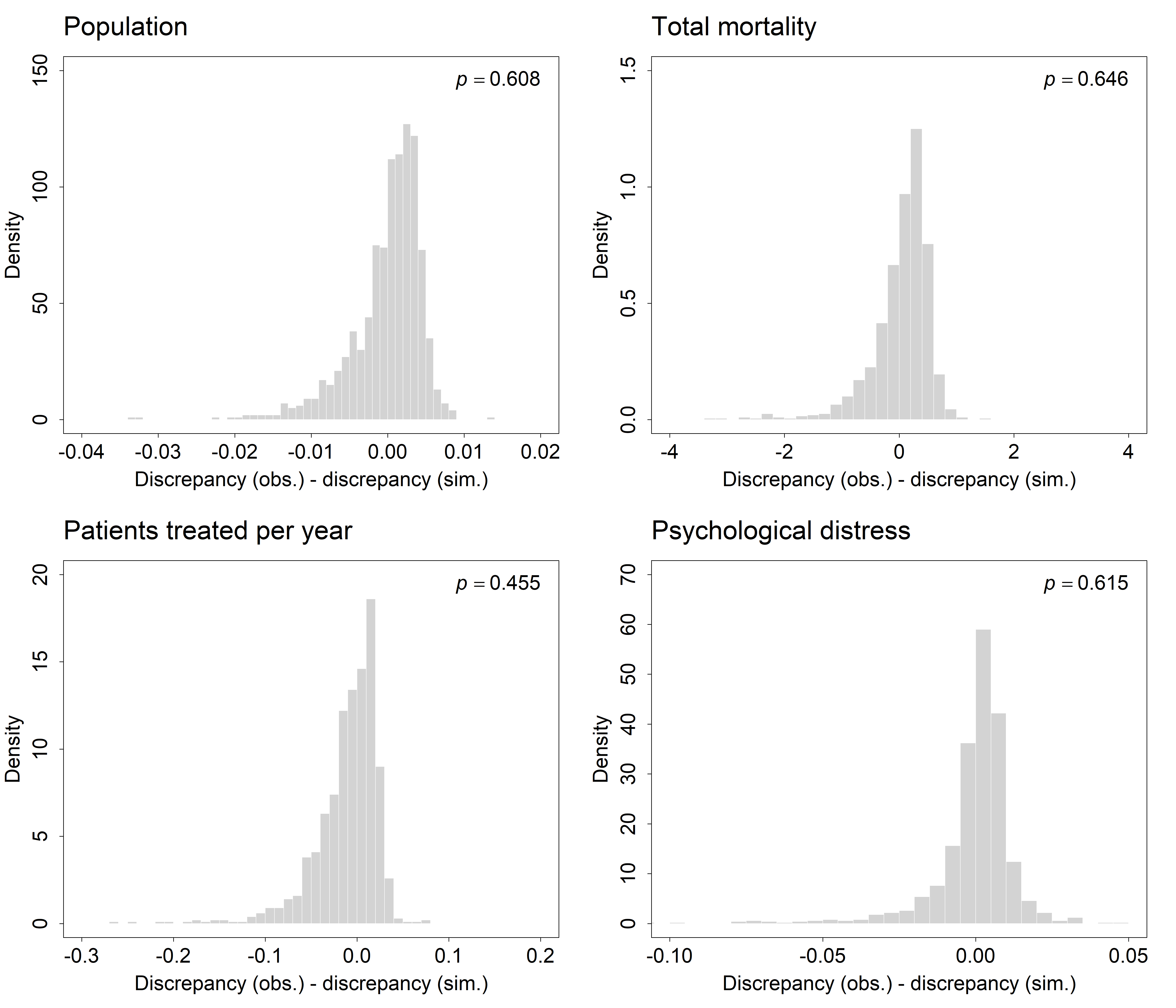


Figure S3. Posterior predictive simulation results for the four data sets used in the analyses (see Table 1 of the paper). Histograms show distributions of the difference between the $\chi^{2}$ discrepancies calculated for the observed data and each of 10^3^ simulated data sets (see Gelman et al., 1996). 95% intervals for all distributions include zero, indicating acceptable model fit (the *p*-values in the top right of each panel are the proportions of the distributions lying above zero).

Supplementary appendix 3

Provision of Medicare-subsidised mental health services in Australia over the period 2008−2019


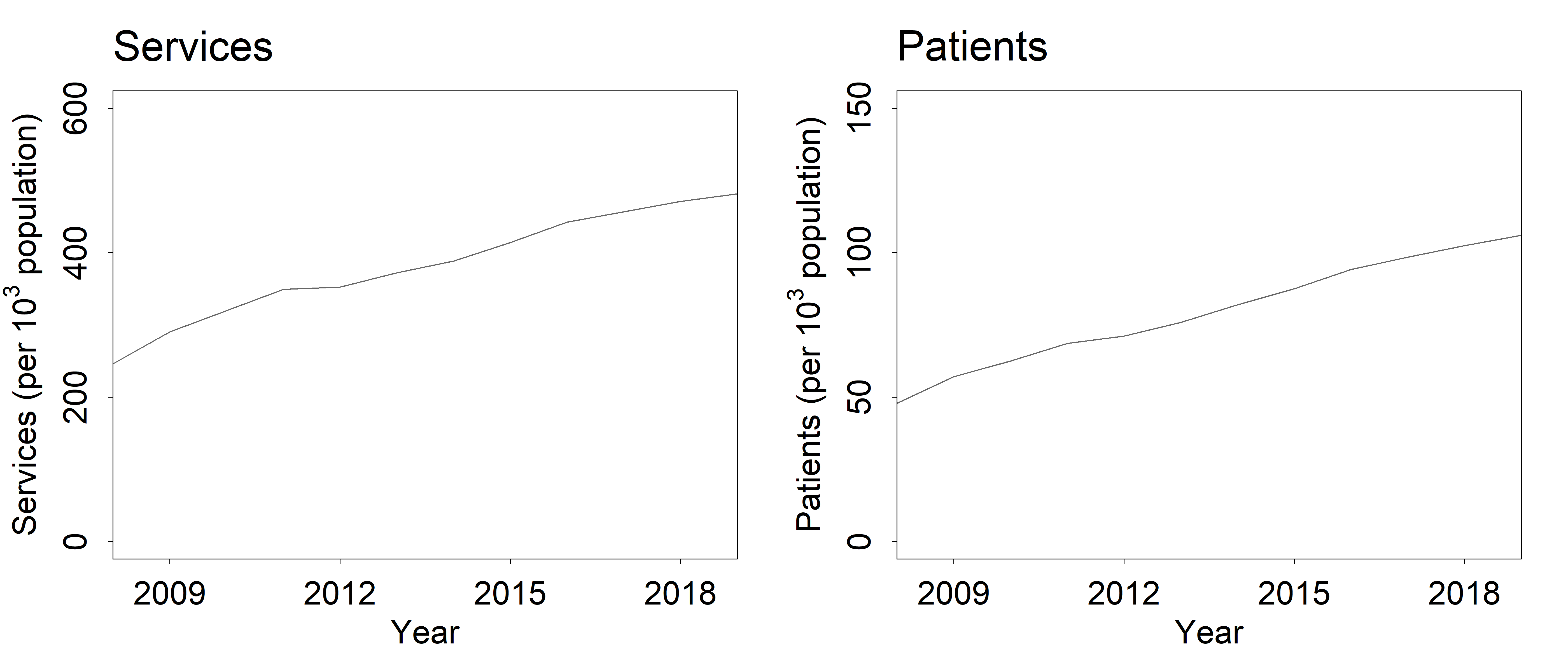


Figure S4. Numbers of Medicare-subsidised mental health services per 10^3^ population per year (left panel) and numbers of patients receiving Medicare-subsidised mental health services per 10^3^ population per year (right panel) in Australia over the period 2008 to 2019 (data are from Australian Institute of Health and Welfare, 2021). Medicare-subsidised mental health services include Australian Government-funded services provided by general practitioners, psychiatrists, and psychologists and other allied health professionals (including social workers, mental health nurses, and occupational therapists) in a variety of settings (consulting rooms, hospitals, at home, or via phone or videoconferencing).

References

Australian Institute of Health and Welfare, 2021. Mental health services in Australia. Medicare-subsidised mental health-specific services 2019−20. Available at: https://www.aihw.gov.au/reports/mental-health-services/mental-health-services-in-australia/data.

Supplementary appendix 4

Results for analyses using psychological distress data from the National Health Survey (cf. figures 2 and 3 of the paper)


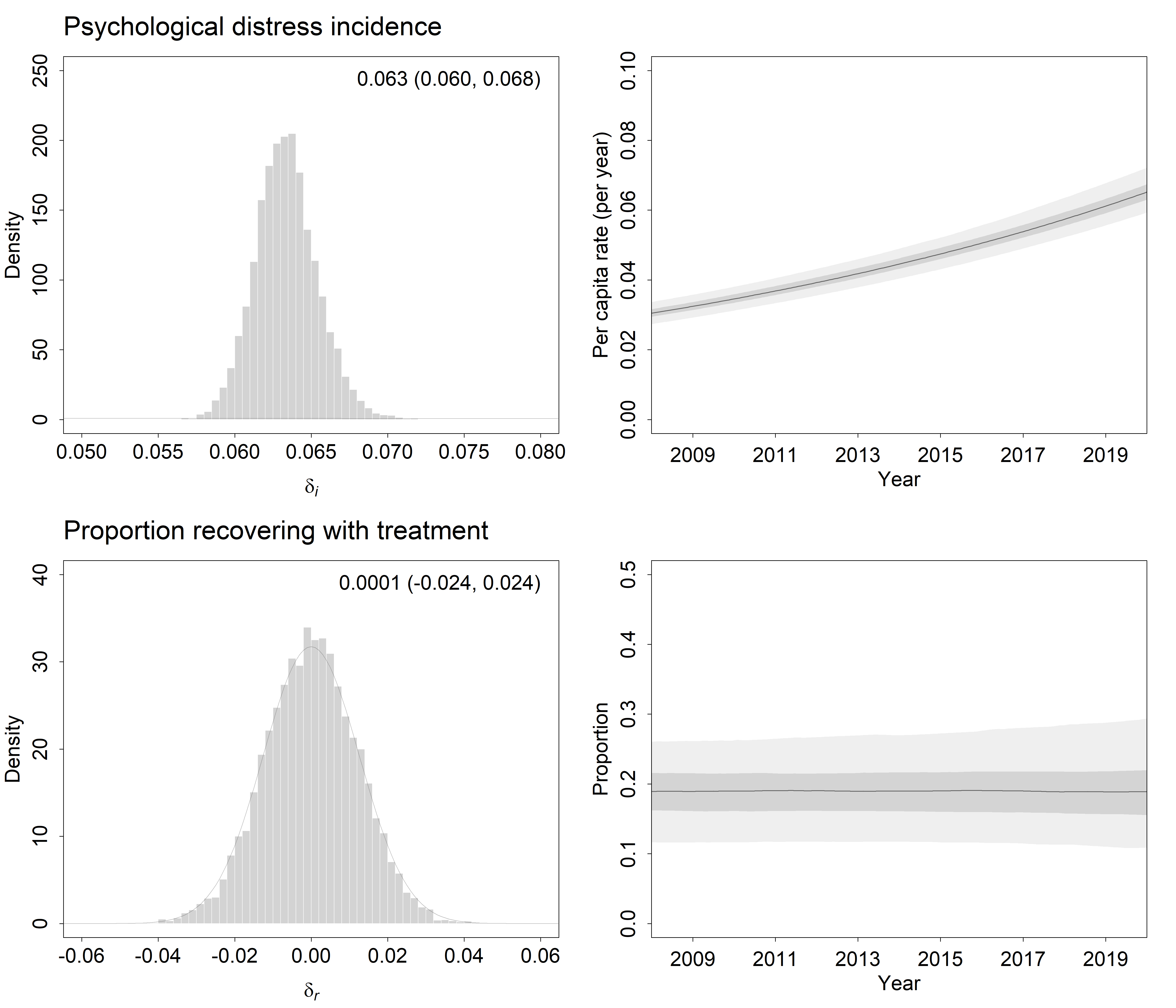


Figure S5. Left panels. Marginal posterior distributions estimated for the fractional rate of increase in the per capita incidence of high to very high psychological distress ($\delta_{i}$) and the fractional rate of increase in the proportion of patients recovering with treatment ($\delta_{r}$). Median estimates and 95% credible intervals are shown in the top right corner of each panel. Prior distributions are plotted as smooth curves. Right panels. Modelled trajectories for the per capita incidence of high to very high psychological distress ($i$) and the proportion of patients recovering with treatment ($r$). Pointwise 50% and 95% credible intervals are indicated with dark grey shading and light grey shading, respectively.


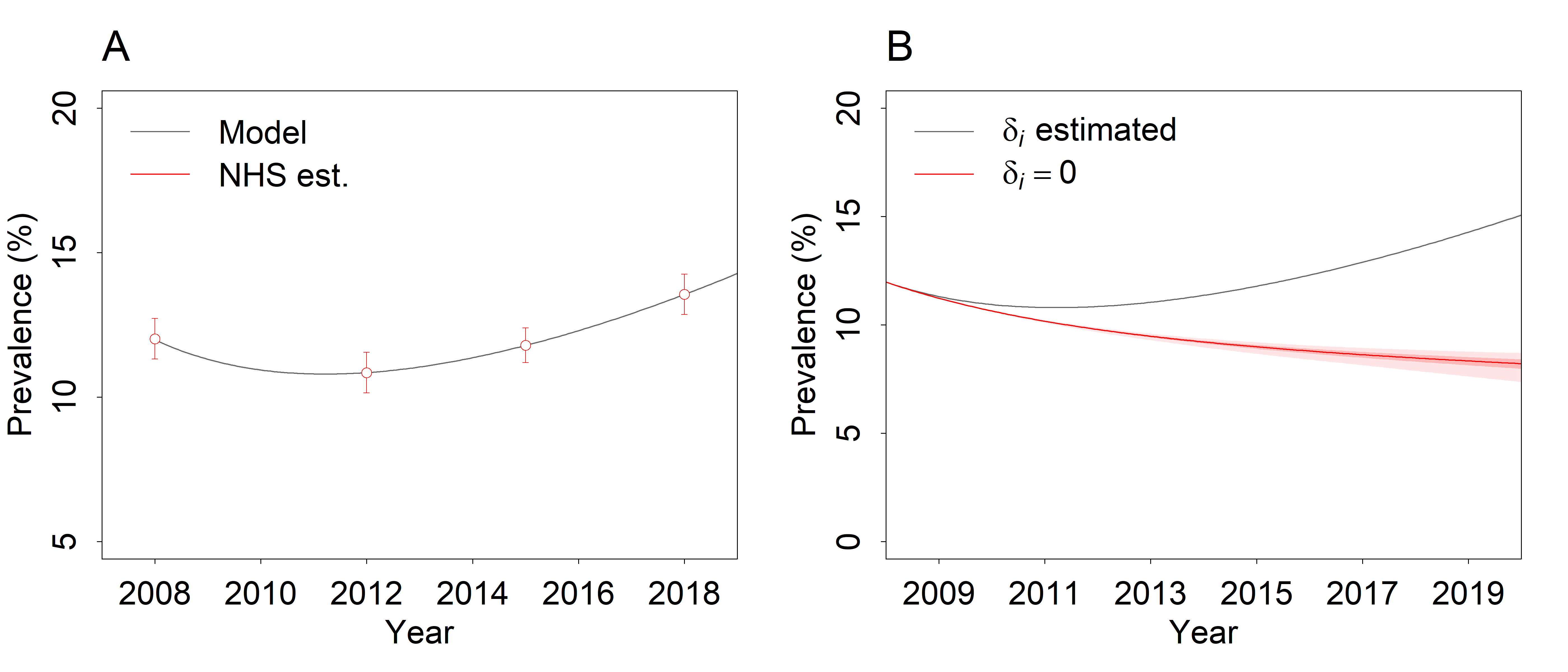


Figure S6. A) Estimates of the prevalence of high to very high psychological distress among Australian adults (18 years and above) over the period 2008 to 2019 derived from the National Health Survey (NHS, red open circles with 95% confidence intervals; Australian Bureau of Statistics, 2018) and the system dynamics model (dark grey line, obtained assuming median parameter estimates). Pointwise 50% and 95% credible intervals are indicated with dark grey shading and light grey shading, respectively. B) Prevalence of high to very high psychological distress simulated under a counterfactual scenario in which per capita incidence remains constant over time ($\delta_{i}$ is set to 0; red line). The model-based estimates from panel A (where $\delta_{i}$ is estimated from the NHS data) are also plotted for comparison (dark grey line). Pointwise 50% and 95% credible intervals are indicated with dark shading and light shading, respectively. Under the counterfactual scenario, the prevalence of high to very high psychological distress decreases by 3.65 percentage points (95% credible interval 3.21−4.34) between 2008 and 2019. Multiplying this prevalence decrease (0.0365) by the adult population in 2019, we obtain an estimate of 714068 fewer people with high to very high psychological distress, which equates to 30.5% of the number of people who would have reported high to very high psychological distress if prevalence had remained constant (i.e., at the 2008 value of 12.0%).

References

Australian Bureau of Statistics, 2018. National Health Survey: first results, 2017−18. Cat. no. 4364.0.55.001. Australian Bureau of Statistics, Canberra.
